# MONTESSORI-BASED APPROACH FOR ALZHEIMER’S AND DEMENTIA: HANDBOOK FOR IMPLEMENTATION IN AN ADULT DAY CARE PROGRAM

**DOI:** 10.1093/geroni/igae098.3867

**Published:** 2024-12-31

**Authors:** Annette Rodrigues

**Affiliations:** California State University, Long Beach, Long Beach, California, United States

## Abstract

Adult day care (ADC) programs serve as an intervention that offers a solution to the challenges faced by an increasing population of individuals living with dementia. Including activities for mental and physical stimulation such as games, exercises, and art, ADC offers dual benefits by providing community-based care for older adults and respite for caregivers. Studies indicate, however, that a lack of quality and standardization negatively impacts the quality of ADC programs, thereby reducing utilization. As program directors expand their services to address the needs of the current demographic, a Montessori approach to dementia care can serve as an effective method of delivering deliberate, evidence-based care. However, most existing applications focus on in-home and long-term care settings rather than ADCs. To address this gap, this project aimed to develop a handbook for implementation in an ADC setting. Montessori-Based Approach for Alzheimer’s and Dementia: Handbook for Implementation in an Adult Day Care Program was developed based on research, observations, and feedback from expert panelists. In the handbook, Montessori principles are presented, barriers and challenges are addressed, organizational strategies are offered, as well as recommendations for developing meaningful person-centered programs, preparing the environment, and addressing participants, staff, and family. By providing a comprehensive guide for an evidence-based standard of care, this handbook aims to advance the field of aging by combining existing research that supports Montessori-based dementia care and facilitating its implementation into an ADC program, with a goal of increasing utilization and improving quality of life.

